# Early electrophysiological diagnosis of ICU-acquired weakness

**DOI:** 10.1186/cc13657

**Published:** 2014-03-17

**Authors:** L Wieske, C Verhamme, E Witteveen, A Bouwes, MJ Schultz, IN Van Schaik, J Horn

**Affiliations:** 1 Academic Medical Center, University of Amsterdam, the Netherlands

## Introduction

An early diagnosis of ICU-acquired weakness (ICU-AW) is difficult because disorders of consciousness preclude strength assessment [1]. Electrophysiological (EMG) studies may be an alternative approach [2]. In this study we investigated feasibility and diagnostic accuracy of EMG studies to diagnose ICU-AW in unconscious patients.

## Methods

Newly admitted unconscious ICU patients (RASS <-3), ventilated for ≥2 days, were included in this single-center prospective cohort study. EMG testing included ulnar (motor/sensory), peroneal (motor) and sural (sensory) studies. Myography was performed when coagulation was normal (dorsal interossei I/II, deltoid and tibial muscles). Reliability of results was checked by an experienced neurophysiologist, blinded for strength. Motor/sensory studies were abnormal if amplitudes were below the 2.5th percentile reference values [3]. Myography was abnormal if spontaneous abnormal activity was found in ≥1 muscles. Upon awakening, strength was assessed (ICU- AW: average MRC <4 [1]), blinded for EMG. Feasibility was determined as the percentage of measurements that could be performed and were reliable. Diagnostic accuracy was analyzed using sensitivity and specificity.

## Results

**Figure 1:**
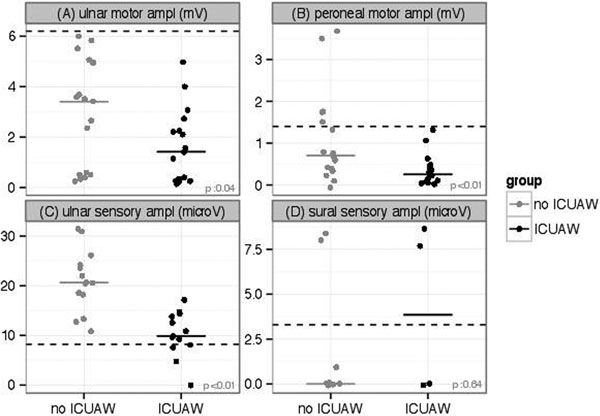
Dotted lines represent 2.5th percentile reference values [3].

We included 35 patients (ICU-AW: 17). EMG testing was done on day 4 (IQR: 3 to 6). Feasibility was 94%, 89%, 77%, 34% and 31% for ulnar motor, peroneal motor, ulnar sensory, sural sensory and myography studies, respectively. Figure 1 displays amplitude values. Sensitivity/specificity was 100%/0%, 100%/31%, 31%/100%, 50%/25% and 67%/38%, respectively.

## Conclusion

Feasibility of ulnar and peroneal studies was acceptable; feasibility of sural and myography studies was low. Diagnostic accuracy was low for all studies. This may be improved with new reference values.
